# Left Atrial Strain, Amy-Lyon, and Columbia Scores to Predict Atrial Fibrillation in Cardiac Amyloidosis

**DOI:** 10.1016/j.jacadv.2026.102879

**Published:** 2026-06-15

**Authors:** Pierre-Yves Courand, Tiphaine Bollon, Xavier Aubertin, Damien Garcia, Pierre Lantelme

**Affiliations:** a Institut de Cardiologie, Hospices Civils de Lyon, Lyon, France; b Université de Lyon, CREATIS, CNRS UMR5220, INSERM U1044, INSA-Lyon, Université Claude Bernard Lyon 1, Lyon, France

**Figure:**
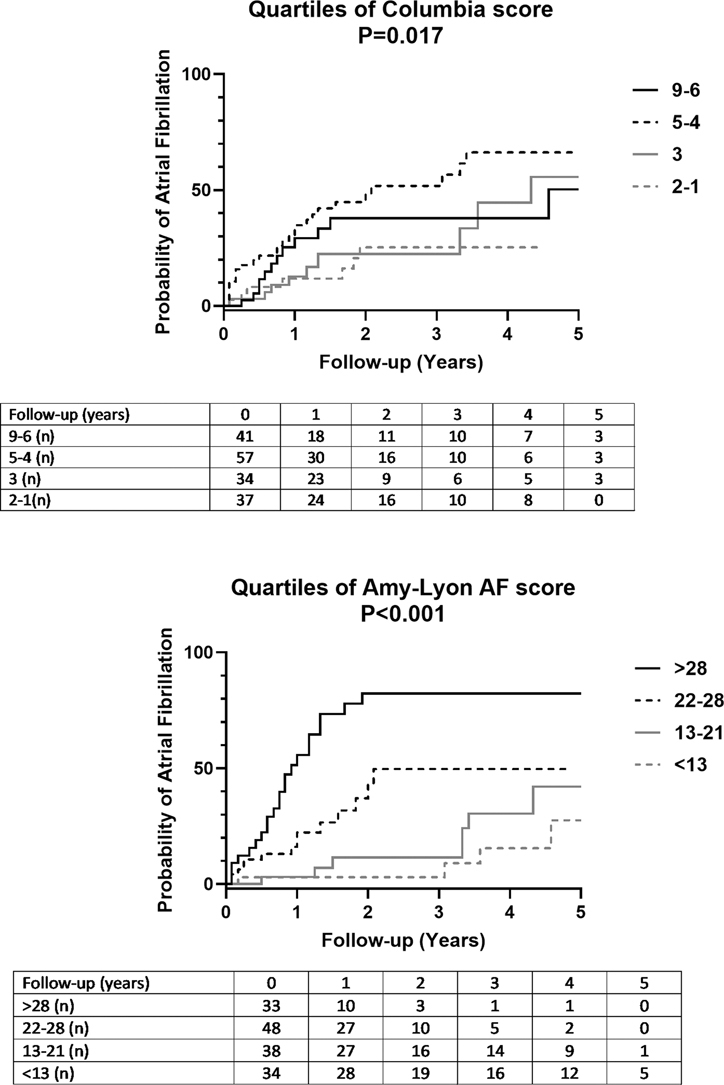

We read with great interest the study by Chan et al^1^, which identified predictors of atrial fibrillation (AF) in transthyretin amyloidosis (ATTR), including amyloidosis type (higher risk in wild-type ATTR), the Columbia score, and tafamidis use. Concurrently, we published data on left atrial reservoir strain^2^ and the Amy-Lyon score—a clinical and electrocardiographic tool comprising hypertension, amyloidosis type, first-degree atrioventricular block, P-wave duration ≥120 ms, and sleep apnea.^3^ Our cohort (n = 169; 56.2% wild-type ATTR, 35.5% light-chain amyloidosis (AL), 8.3% variant ATTR) included patients in sinus rhythm at diagnosis, with 55 (33%) developing AF over a median follow-up of 21 months.^3^

**Figure 1:**
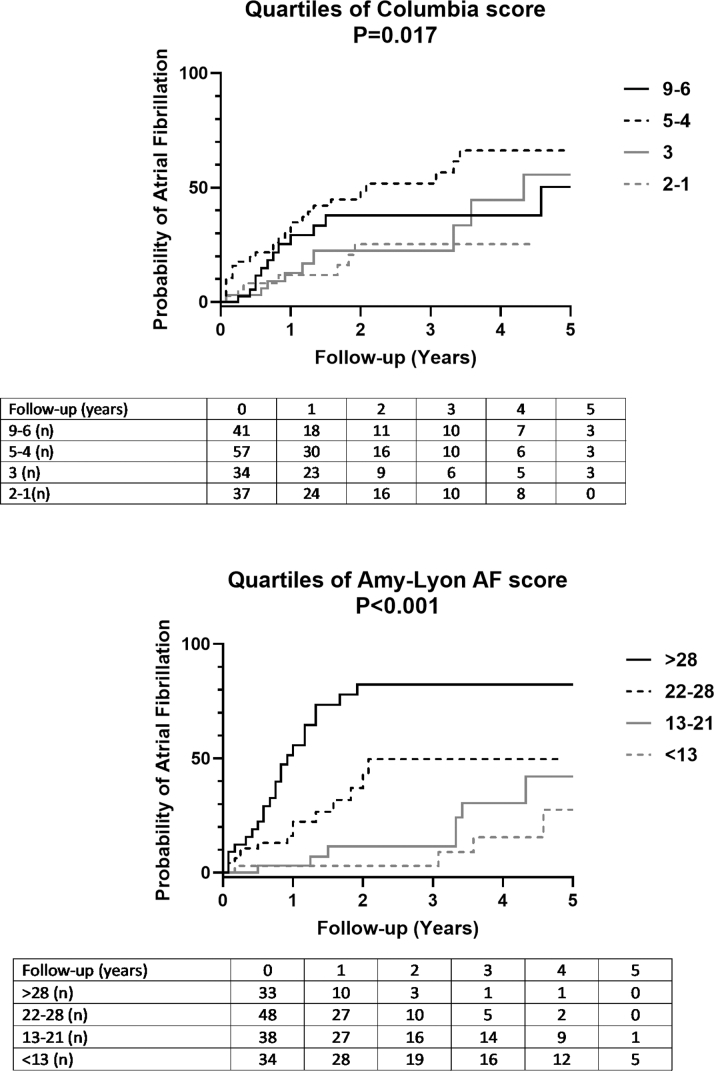
Incidence of Atrial Fibrillation Upper panel: probability of incident atrial fibrillation according to quartiles of Columbia score Lower panel: probability of incident atrial fibrillation according to quartiles of Amy-Lyon AF score Quartiles of Columbia score: 1 to 2, first quartile; 3, second quartile; 4 to 5, third quartile; 6 to 9, fourth quartile. Quartiles of Amy-Lyon AF score: <13, first quartile; 13 to 21, second quartile; 22 to 28, third quartile; >28, fourth quartile. *P* value for log rank. AF = atrial fibrillation.

In the overall cohort, the Amy-Lyon score outperformed the Columbia score in predicting incident AF, with area under the curve (AUC) values of 0.768 (0.685–0.852) (*P* < 0.001) and 0.593 (0.504–0.681) (*P* = 0.049), respectively (*P* for comparison = 0.150). In the ATTR subgroup (n = 109), the Amy-Lyon score’s AUC was 0.804 (0.707–0.902) (*P* < 0.001) vs 0.615 (0.507–0.724) (*P* = 0.047) for the Columbia score (*P* for comparison = 0.193). In the AL subgroup (n = 60), the Amy-Lyon score’s AUC was 0.756 (0.625–0.887) (*P* = 0.004), compared to 0.597 (0.447–0.747) (*P* = 0.252) for the Columbia score (*P* for comparison = 0.425). Among patients with available strain data (n = 84), AUCs were 0.754 (0.653–0.855) (*P* < 0.001) for left atrial reservoir strain, 0.768 (0.660–0.877) (*P* < 0.001) for the Amy-Lyon score, and 0.642 (0.518–0.765) (*P* = 0.013) for the Columbia score. Kaplan-Meier analyses using quartiles of both scores confirmed their association with AF, although the Columbia score showed a less distinct gradation (Figure 1, *P* using log-rank test). Our results externally validate the Columbia score in our cohort. The differences in predictive performance may relate to our cohort’s composition, particularly the inclusion of AL amyloidosis patients.

We congratulate Nicholas Chan et al on their work. We would be interested to know if their team has corresponding data to evaluate the performance of left atrial strain or the Amy-Lyon score parameters in their ATTR cohort, which could help clarify the generalizability of these different predictive tools.
